# Bibliometrics, the "Clinical Diagnostics" of Research Impact: Comment on "‘We’re Not Providing the Best Care If We Are Not on the Cutting Edge of Research’: A Research Impact Evaluation at a Regional Australian Hospital and Health Service"

**DOI:** 10.34172/ijhpm.2023.7703

**Published:** 2023-05-02

**Authors:** Giovanni Abramo, Ciriaco Andrea D’Angelo

**Affiliations:** ^1^Laboratory for Studies in Research Evaluation, Institute for System Analysis and Computer Science, National Research Council of Italy, Rome, Italy; ^2^Department of Engineering and Management, University of Rome “Tor Vergata,” Rome, Italy

**Keywords:** Evaluative Bibliometrics, Research Evaluation, Research Impact, Research Management

## Abstract

Following the Townsville Hospital and Health Service (THHS) strategic revision as a "research-based" institution, Brown et al have investigated the impact of THHS research, and its key drivers, based on 15 stakeholder interviews and two quantitative indicators. This commentary argues that the quantitative analyses and findings would have benefitted from applying evaluative bibliometrics, hopefully, conducted by experienced bibliometricians. We present the potential of bibliometrics for assessing the scholarly impact of research, as well as a few examples of its application to the case of THHS, for informing research policies and strategies.

 Admirably, Brown et al^1^ have recently set out to evaluate the impacts of research investments and the key drivers in one of the regional health services composing the Australian national system. Justly according to their title, “*We’re not providing the best care if we are not on the cutting edge of research* …^.^”

 Although this is a case study dealing solely with health research, in one part of one state of one nation, the problems addressed by these authors, the questions they ask, and the limitations they encounter in seeking answers seem exemplary of those arising in evaluating the results from public research organisations and systems around the world. Indeed the concerns and complications addressed by Brown et al are not limited to medical research but extend to the higher education systems of entire nations, forged on the principle that effective teaching descends from qualified research, also known as the “Humboldtian model.”^2^ In fact, in the global context, the universities most attractive to potential students and faculty are precisely those also the world leaders in research. And in the current knowledge-based economy, it is the nations on the cutting edge of research that are also most capable of sustaining vigorous socio-economic development.

 In general, the solutions to the growing challenges and problems of global warming, energy sustainability, health and nutrition for populations, income inequality and scarcity of resources, demand: (1) increasing efficiency in our research systems; (2) care in allocating public funds for research; and (3) speeding up the cross-sector transfer process.

## Evaluation of Research Impact

 Public investment cannot take place in an information vacuum. The evaluation of the impacts achieved is essential for the development and refinement of the research policies of nations and regions, and the same for the research strategies of individual organisations. Knowing the research strengths and weaknesses of territories and organisations, the “public investor” can then intelligently direct the continuing allocation of funds. Evaluation, united with performance-based incentive schemes, stimulates the research productivity of organisations and individuals. Companies, students, patients, etc need information on the capabilities of the research organisations and researchers they seek: evaluation reduces the problems of “information asymmetry” in demand and offer. Not to be forgotten, from the communication of assessments, citizens learn that the investments of their tax funds are effectively used in producing benefits.

 Accepting that the evaluation of the impact of research activity is essential, we must also understand the two main types of impact: scholarly and social.^3^ “Scholarly impact” refers to the contributions from research in further advancements of knowledge, ie, considering solely the impacts within the sector of the scientific community. “Social impact” instead refers to the contributions from research in the social application beyond the strictly scientific sectors,^4-6^ eg, from research in the medical fields, the adoption of new health protocols, the manufacture of new medical equipment, or the distribution of new vaccines.^7^ Compared to any scholarly impacts, which will be relatively quick, social impacts might take years or even decades to materialise,^8^ often resulting from sequences and combinations of scholarly impacts, making their evaluation much more difficult. Achieving social impacts requires participation from the actors of production systems and public institutions far beyond the scholarly world. In any case, it is clear that for research to have an impact, the results must finally be used: zero use equals zero impact; an invention that is never used brings no social benefits,^9^ and scientific publications that are never read and cited by other scholars have no scholarly impact.

 We have witnessed in recent years a growing attention of policy-makers to the evaluation of social impact and, alongside, a growing attraction of evaluative bibliometricians to the investigation of metrics alternative (altmetrics) to citation-based ones. Typical altmetrics are manuscript pageviews or downloads, and mentions on social networks, but none of them certifies real use and, therefore, can be considered a reliable proxy of social impact. The only possible exceptions are the references in public policy documents; or commentary from experts and practitioners. In Woolston’s words: “Approaches to capturing the benefits of research on society are improving — but huge challenges remain.”^10^ Our personal view is that when a timeliness research assessment is critical, as is always the case when it has to inform strategic and policy decisions, the scholarly impact remains the most reliable proxy of social impact.

## Evaluative Bibliometrics

 Brown et al base their quantitative evaluation of the research impact at Townsville Hospital and Health Service (THHS) on two indicators: (1) the number of site-specific approvals (SSAs) for research, which tripled between 2010 and 2018; and (2) the number of publications, which increased by 17% between 2015 and 2018. They conclude, “These increases are likely to reflect greater clinician engagement in research over time.” The authors also note, however, that they had scarce access to relevant information, which hampered their analyses and recommend establishing internal data-collection systems. One could imagine, though that the establishment and operation of data information systems would be costly in funds and staff time, especially if this were to draw clinicians into the provision and updating of new information and so away from their heavily tasked core roles, or indeed demand the hiring of specialised personnel. To minimise the burden on THHS clinicians and budgets, it would seem attractive to instead search for other avenues of accurate and reliable impact measurement based on already existing databases, and for the THHS to seek external specialists for outsourcing such data, as well as their subsequent processing.

 Departing from the two indicators measured by Brown et al in this subsection, we aim to guide the reader toward the potential of bibliometrics in the evaluation of the scholarly impact of research. As we have often heard, “not everything that can be counted counts, and not everything that counts can be counted,”^11^ and in fact, the number of SSAs for research fails to inform on what increase there may have been in THHS research investments. The average internal and external funds allocated to each SSA, as an example, may have become less. Nor does an increase in the measure of the number of publications necessarily correspond with increasing scholarly impact: again, there may have been a decrease in the average impact of these publications; the average contribution of THHS authors to extra-mural multi-author publications may have decreased; an increase in publications may have no corresponding increase in the number of highly cited articles, eg, in the top x% of world rank by the number of citations. Most importantly, we do not know whether research productivity has improved at the THHS, in terms of impact per A$ spent on research, nor whether THHS invests more in the research fields (specialization indexes) where it excels.

 Nor are we informed on the areas of THHS research strengths and weaknesses relative to national or international benchmarks. We do not even know the institution’s overall scientific standing; certainly not the productivity rank of individual THHS researchers in respect of benchmarks. Could there be fields where the THHS has national or world top scientists? Are THHS researchers tending more to specialise or diversify their fields of investigation, and are they conducting more or less interdisciplinary research? If research collaborations have increased (intramural, and especially domestic extramural, international, and cross-sector), then what institutions and countries have THHS found most productive for establishing such collaborations? In general, how does the THHS figure in the balances of citing vs cited publications, ie, national and global flows of knowledge? Finally, what about details of effectiveness in researchers’ recruitment and turnover, or the possible gender differences in research activity and productivity? Are these matters on which the administration, and indeed the existing personnel, should be informed?

 The administrators of the THHS and Australian health system can have answers to all the above questions, more or less precise and reliable, through bibliometrics, loosely defined as the entire set of methods for quantitative analysis of scientific and technological literature.^12^ In particular, evaluative bibliometrics, first introduced by Narin,^13^ is the application of bibliometrics for the evaluation of scientific activity, especially scientific performance. Evaluative bibliometrics builds on two pillars of information: (1) publications indexed in bibliographic repertories, as a measure of research output; and (2) citations received, as a measure of their value, called by bibliometricians “scholarly impact.” The underlying rationale is that, for research results to have an impact they have to be “used,” and citations certify their use.^14^ The intrinsic limits of evaluative bibliometrics are apparent: (1) publications are not representative of all knowledge produced (tacit knowledge is not captured); (2) bibliographic repertories do not cover all publications; and (3) citations are not always certification of real use nor representative of all uses, hence, the great effort made in recent years to develop altmetrics. Nonetheless, bibliometrics outperforms peer review in predicting the scholarly impact of research activity.^15^

## Scholarly Impact of THHS Research

 This subsection gives examples of the application of evaluative bibliometrics for the assessment of THHS research activity and its scholarly impact. Given the space and especially desire for timely comment on the original Brown et al article, we report only key findings at the aggregate level, obtained without access to any new data from THHS. Publications by institutionally based authors were instead extracted from the Web of Sciences Core Collection. The period under observation is 2003-2021, reflecting the original aim of assessing the changes in impact after the 2008 THHS strategic revision as a research-based hospital and health service.

 Results in Figure show a slight increase in the number of publications from 2008 onwards, which becomes more pronounced from 2012, when publications doubled in two years. The average normalised impact of each publication (the number of citations of a publication normalised to the average number of citations of the world publications of the same year and same Web of Science Core Collection subject category) has however decreased. Still, overall, the total normalised impact (sum of the normalised impact of all publications) has been increasing. Interestingly the total number of top 10% cited publications by THSS staff has been increasing. Finally, research activity in collaboration with foreign institutions has also increased. Access to the names of THHS clinicians would have allowed higher precision in the analyses.^16^

**Figure:**
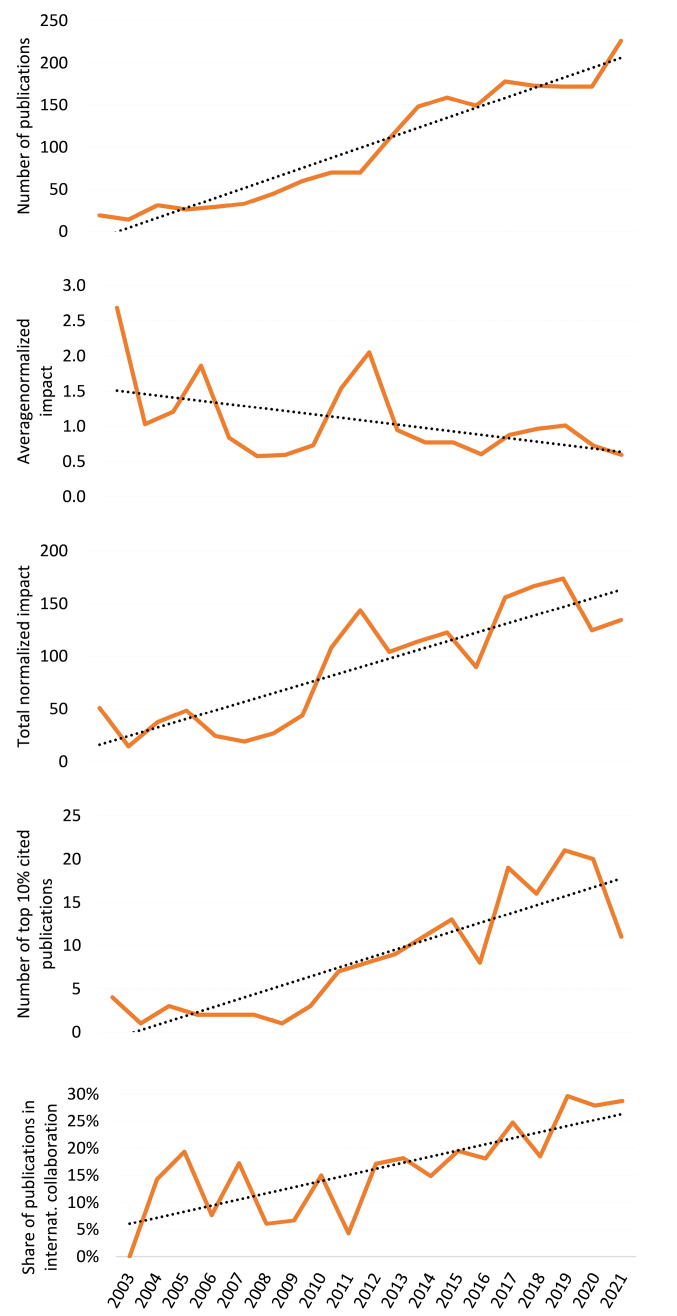
Townsville Hospital and Health Service Research Activity and Impact Indicators, 2003-2021. Data source: Web of Science Core Collection.

 What should come next, for institutional and national health administrations, is the calculation of productivity, ie, ratio of total normalised impact to A$ spent on THHS research, but this measurement would demand data on research expenditures, to be supplied by the administrations themselves.

 We hope that this brief commentary will illustrate the practical potentials of evaluative bibliometrics, available at a relatively low cost, versus the risks of arriving at dead ends on the road of do-it-yourself evaluations. In fact, the combination of bibliometric analyses with the qualitative surveys and interviews conducted by Brown et al would result in a powerful strategic analysis that could truly benefit the management of research institutions. We hope that as clinicians continuously advance in the selection and use of diagnostic instruments, accepted without fear by patients, research policy-makers and managers should also bravely advance in the selection of their own diagnostic tools, in particular without fear of bibliometrics.

## Ethical issues

 Not applicable.

## Competing interests

 Authors declare that they have no competing interests.

## Authors’ contributions

 Conceptualization: Giovanni Abramo and Ciriaco Andrea D’Angelo.

 Data curation: Ciriaco Andrea D’Angelo.

 Investigation: Giovanni Abramo and Ciriaco Andrea D’Angelo.

 Methodology: Giovanni Abramo and Ciriaco Andrea D’Angelo.

 Supervision: Giovanni Abramo.

 Visualization: Ciriaco Andrea D’Angelo.

 Writing–original draft: Giovanni Abramo and Ciriaco Andrea D’Angelo.

 Writing–review & editing: Giovanni Abramo.
